# An information-based network approach for protein classification

**DOI:** 10.1371/journal.pone.0174386

**Published:** 2017-03-28

**Authors:** Xiaogeng Wan, Xin Zhao, Stephen S. T. Yau

**Affiliations:** Department of Mathematical Sciences, Tsinghua University, Beijing, China; Harbin Institute of Technology Shenzhen Graduate School, CHINA

## Abstract

Protein classification is one of the critical problems in bioinformatics. Early studies used geometric distances and polygenetic-tree to classify proteins. These methods use binary trees to present protein classification. In this paper, we propose a new protein classification method, whereby theories of information and networks are used to classify the multivariate relationships of proteins. In this study, protein universe is modeled as an undirected network, where proteins are classified according to their connections. Our method is unsupervised, multivariate, and alignment-free. It can be applied to the classification of both protein sequences and structures. Nine examples are used to demonstrate the efficiency of our new method.

## Introduction

The protein universe is diverse for sequences, structures and functions, and classification of sequences may infer the structures and functions of proteins. Therefore, it is important to analyze the taxonomy of proteins. Many methods have been developed to classify proteins [1–14]. Among the various methods, many compute the biological distances and classify proteins using polygenetic-trees, since sequence and structural similarity is considered to be closely related to protein homology [2]. Some representatives of these methods are the natural vector method [10, 13, 14], protein map [8,9], K-string dictionary [11], and Yau-Hausdorff distance [6]. These methods are unsupervised that do not require the usage of labeled training data. For supervised approach in machine learning, support vector machine classifiers/predictors are often used [15–23]. LibSVM, developed by Chang and Lin [15], is a library for support vector machines that has gained wide popularity in machine learning and many other areas [15]. LibD3C is an ensemble classifier which is based on clustering and parallel implementation [17]. nDNA-Prot proposed in [22], is a new predictor to accurately identify DNA-binding proteins when combines with an ensemble classifier. Also, an improved protein structural classes predictor is proposed in [23] which incorporates both sequence and structure information. Another novel techniques [20] is proposed to impressively increase the accuracy of protein fold classification. Moreover, a multi-objective evolutionary algorithm, bases on membranes [19], is invented to solve the network clustering problem. Furthermore, a new predictor, named iDHS-EL, is proposed by using the strategy of ensemble learning framework [21], to identify the location of DHS in human genome. These supervised classification methods largely depend on the usage of labeled training data, but cannot be directly used on unlabeled test data.

Early protein classification methods concern only the bivariate relationships of proteins [2–4, 6, 9–14]. However, these bivariate relationships do not fully reflect the true genetic relationship of proteins [19]. Typically, the protein universe is complex and diverse, and proteins are more likely to have multivariate relationships than bivariate relationships [19]. In other words, one protein may be simultaneously related to many other proteins. To form a natural picture of the protein universe, theories of information and networks [24,25] are used to develop new methods that survey the multivariate relationships of proteins [26].

In this paper, we propose a new method on protein evolutionary classification. The organization of the paper is as follows. In section of Materials and methods, mathematical theories of the maximum mutual information rates and background of connected component are introduced. In the Results section, nine examples, including both sequence and structure examples, are presented to demonstrate the implementation and usefulness of our method. In the Discussion section, the application of the nine examples are discussed, where advantages, disadvantages, and the differences between our method and other methods are also summarized.

## Materials and methods

The new method is based on theories of information and networks. It begins with the discrete map of amino acid sequences; the mathematical details of the new method are described as follows.

### The maximum mutual information rates

We analyze the amino acid sequence data of the proteins. A protein sequence is made up of 20 possible types of amino acids; and it can be viewed as a discrete time series with 20 possible states. Each amino acid *a* ∈ {*A*,*C*,*D*,*E*,*F*,*G*,*H*,*I*,*K*,*L*,*M*,*N*,*P*,*Q*,*R*,*S*,*T*,*V*,*W*,*Y*} can be uniquely mapped to an integer *b* ∈ {1,2,3,4…,20}. Via this mapping, the whole sequence is transformed into a discrete time series.

For a pair of integer time series *X* and Y, with length M and N respectively (*M* ≤ *N*), define Y_*i*_ (1 ≤ *i* ≤ *N* − *M* + 1) as the i-th length M segment of Y; the mutual information rate [26–30] between X and Y_*i*_ (1 ≤ *i* ≤ *N* − *M* + 1) is calculated as:

$$I(X;Y_{i})=\sum_{\alpha \in S_{X},\beta \in S_{Y_{i}}}p(x=\alpha ,y=\beta )\log\frac{p(x=\alpha ,y=\beta )}{p(x=\alpha )p(y=\beta )},$$ (1)

where *S*_*X*_ and *S*_*Yi*_ stand for the state sets (subsets of integers from 1 to 20) of X and Y_*i*_ respectively. Note that the summation of this equation is position-free, it sums over all combinations of the states of X and Y_*i*_. The mutual information rate is the probability expectation of log ratios, it depends only on the probability distribution of the states rather than the value of the states.

Varying *i* from 1 to *N* −*M* +1, we get a series of mutual information rates: *I*_1_,…, *I*_*N*−*M*+1_. We take the maximum of the mutual information rates for sequences X and Y:

$$I_{XY,\max}=\underset{1\leq i\leq N-M+1}{\max}I(X;Y_{i})$$ (2)

The maximum mutual information rates are calculated for each pair of the integer sequences. We get a matrix for the maximum mutual information rates, where *I*_*XY*_ is the (X,Y)-th element of the matrix.

The mutual information rate describes the mutual relations between sequences. The maximum mutual information matrix is used as the adjacency matrix to model the protein network. In the protein network, each protein is a node, and different nodes are connected by their mutual relations. The protein network is a weighted network, with the adjacency matrix as the weights. The protein network is undirected [24] because the mutual information rate is symmetric [30]:

$$a_{XY}=a_{YX}=I_{XY,\max}=I_{YX,\max}.$$ (3)

For two arbitrary sequences X (length M) and Y (length N) in the dataset, the maximum mutual information rate *I*_*XY*,max_ is reached for some *k*, 1 ≤ *k* ≤ *N* − *M* + 1:

$$I_{XY,\max}=I(X;Y_{k}).$$ (4)

Because in information theory the mutual information rate is upper bounded by the entropies [26–30], we have:

I(A;B)≤min{H(A),H(B)}, (5)

where $H(A)=-\sum_{\alpha \in S_{A}}p(a=\alpha )\log\;p(a=\alpha )$, $H(B)=-\sum_{\beta \in S_{B}}p(b=\beta )\log\;p(b=\beta )$, A and B denote two general sequences of the same length. Therefore, we have

$$I_{XY,\max}=I(X;Y_{k})\leq \min\{H(X),H(Y_{k})\}.$$ (6)

In addition, *Y*_*k*_ is a sub-sequence of *Y*, and we therefore have [30]:

$$H(Y_{k})\leq H(Y),$$ (7)

and the inequality holds:

$$I_{XY,\max}\leq \min\{H(X),H(Y)\}.$$ (8)

To make a universal comparison between the nodes, the adjacency matrix is normalized by the maximum entropy. Assume there are N proteins in the database; the adjacency element between the *i*th and the *j*th proteins becomes:

$$a_{ij}=\frac{I_{ij,\max}}{\underset{1\leq q\leq N}{\max}H_{q}}$$ (9)

where *H*_*q*_ denotes the entropy of the *q*th protein. Elements of the adjacency matrix are bounded between 0 and 1.

### Connected component

For the normalized adjacency matrix, a mutative threshold is defined to filter the adjacency matrix. The mutative threshold is defined as a constant multiple of the maximum adjacency element, i.e.,

$$T_{c}=c\cdot \underset{i,j}{\max}\;a_{ij}$$ (10)

where *c* is the multiplicity that varies between 0.1 and 1. For each multiplicity *c*, *T*_*c*_ is used to filter the adjacency matrix, and elements below the threshold are set to zero, where others are unchanged. For the filtered adjacency matrix, the connected components are obtained by examining the adjacency elements. In matrix form, the filtered adjacency matrix takes powers with power s from 1 to N-1 (N stands for the number of nodes in the network, which is also the size of the adjacency matrix). In a network, the connected components are the maximum sets of nodes, where the members are mutually connected to each other by at least one path [4]. To obtain the connected components, the power s adjacency matrix is summed from s = 1 to s = N-1:

$$A_{sum}=\sum_{i=1,\cdots ,N-1}A^{i}$$ (11)

The connected components are the maximum sets of nodes with all elements being positive in the sum matrix *A*_*sum*_; adding new nodes to the components will break this property. Intuitively, by reversible transformations, the sum matrix *A*_*sum*_ can be transformed into block diagonal form:

$$A_{sum}=\left(\begin{array}{ccc} D_{1} & 0 & \cdots \\ 0 & D_{2} & \cdots \\ \vdots & \vdots & \ddots \end{array}\right),$$ (12)

where *D*_*i*_ (*i* = 1,2,⋯) is a positive diagonal matrix corresponding to the connected components of the network.

Varying the multiplicity c, the connected components are changed. The connected components of a higher threshold have stronger connections between their nodes than the components of a lower threshold. Consequently, the components of a higher threshold are included by the components of a lower threshold. We create the protein classifications according to the inclusion/exclusion of the connected components.

## Results

In this section, we use nine examples to demonstrate the efficiency of our method. Graphs of connected components are presented to illustrate the classification analysis.

### Mitochondrial proteins of 28 mammal species

In the first example, we analyze the mitochondrial proteins of 28 mammal species. The dataset is a sample dataset for protein classification analysis [1,9,31,32]. The dataset is made up of 28 proteins encoded by the mitochondrial genome of 28 different mammal species [1,9,31,32]. Each sequence is concatenated from 10 proteins (COI, COIII, COII, Cyt-b, ND1, ATPase 6, ND4, ND5, ND6, ND2) encoded by the same strand of the mitochondrial genome [1,9,31,32]. Among the 13 protein-coding mitochondrial genes, the 3 shortest genes (ATPase 8, ND3, and ND4L) are excluded, and the 10 proteins (COI, COIII, COII, Cyt-b, ND1, ATPase 6, ND4, ND5, ND6, ND2) are coded by the 10 genes that remain [1,9,31,32]. The Genbank accession number of the 28 mitochondrial proteins are the Hedgehog (GenBank accession number X88898), Mouse (J01420), Rat (X14848), Cat (U20753), Gray seal (X72004), Harbor seal (X63726), Horse (X79547), Donkey (X97337), Rhinoceros (X97336), Cow (V00654), Fin whale (X61145), Blue whale (X72204), Gibbon (X99256), Sumatran orangutan (X97707), Bornean orangutan (D38115), Gorilla (X93347), Pygmy chimpanzee (D38116), Chimpanzee (D38113), Human (X93334), Tiger (EF551003), Dog (U96639), Wolf (EU442884), Black bear (DQ402478), Brown bear (AF303110), Polar bear (AF303111), Opossum (Z29573), Wallaroo (Y10524), and Platypus (X83427).

**Fig 1:**
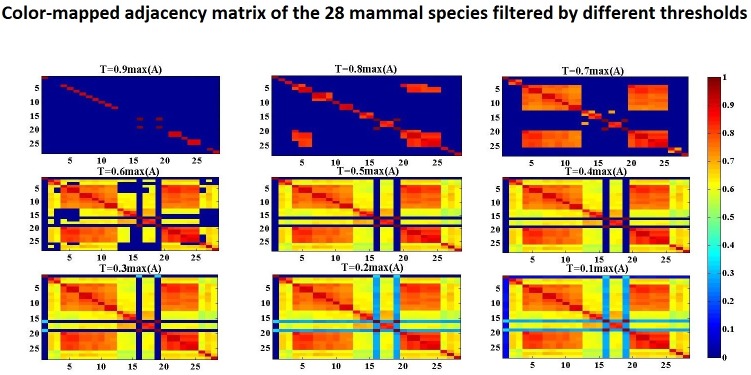
Color-mapped adjacency matrix of the 28 mitochondrial proteins of mammal species. This figure shows the color-map of the filtered adjacency matrices for the 28 mitochondrial proteins of mammal species. The multiplicity c of the filtering threshold (*T*_*c*_ = *c* •*A*_*max*_) is varied from 0.9 to 0.1. The elements of the adjacency matrices are mapped to colors from cold (dark blue, minimum connectivity value) to warm (dark red, maximum connectivity value) as indicated in the color bar.

A color-map of the adjacency matrix with different filtering thresholds is shown in Fig 1. In this figure, the whole adjacency matrix is mapped to colors as shown in the color-bar. The color ranges from cold (blue) to warm (red), indicating the value of the adjacency elements from the minimum to the maximum. Two nodes are considered in one connected component if their adjacency element is positive; this corresponds to a bright color in the color matrix. From the figure, we can see that the color matrix with a higher threshold has fewer bright areas than the color matrix with a low threshold. This indicates that the connected components with a higher threshold are smaller than the connected components with a lower threshold.

**Fig 2:**
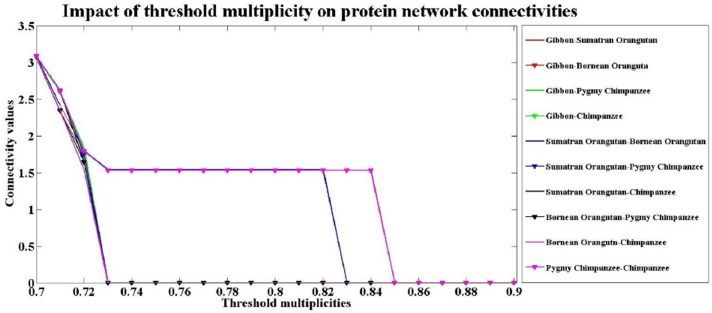
Impact of the threshold on protein network connectivity. This figure shows the line plots of the connectivity values against different multiplicities of the threshold (0.7 ≤ *c* ≤ 0.9). The connectivity value is expressed as $\bar{a}(i,j)=\frac{\sum_{n=1,2,\ldots ,27}a_{n,ij}^{1/n}}{27}$, where 27 is the number of the powered adjacency matrices *A*^*n*^ in series, and n is the power from 1 to N-1 (N = 28 is the number of nodes in the network), and *a*_*n*,*ij*_ is the *ij*-th element of the power *n* adjacency matrix *A*^*n*^. Nodes i and j are considered in the same connected component if $\bar{a}(i,j)>0$. This graph presents a sample analysis of five primate species (Gibbon, Sumatran Orangutan, Bornean Orangutan, Pygmy Chimpanzee and Chimpanzee). The multiplicity *c* is varied from 0.7 to 0.9.

To demonstrate the influence of the threshold, a sample analysis is performed for the 5 primate species (Gibbon, Sumatran Orangutan, Bornean Orangutan, Pygmy Chimpanzee and Chimpanzee). The connectivity values for different thresholds are shown in Fig 2. In this figure, we can see the trends in the connectivity values between the species. Two species are considered in one connected component if their connectivity value is positive. For instance, the Pygmy chimpanzee and Chimpanzee are in the same connected component when *c* = 0.84 (pink line). The Sumatran Orangutan and Bornean Orangutan are connected and made up of a connected component when *c* = 0.82 (blue line). The five primate species are all included in one component when *c* = 0.72. We can see that the connected components are merged together when the threshold multiplicity decreases.

**Fig 3:**
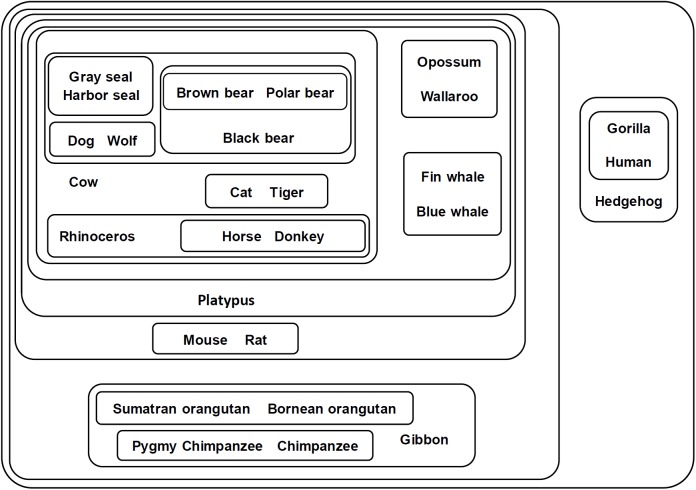
Component graph of mitochondrial proteins of 28 mammal species. This figure shows the connected components of 28 mitochondrial proteins of different mammal species. Each set represents a connected component obtained from the adjacency matrix filtered by a certain threshold (*T*_*c*_ = *c*•*A*_*max*,_ *c*∈[0,1]). Components with higher thresholds are included with components with lower thresholds. The proteins are represented by their mammal species.

The classification result is shown in Fig 3. In this figure, the mitochondrial proteins of 28 mammal species are classified by the connected components shown by the contours. Each protein is represented by the name of its mammal species; e.g., the mitochondrial protein of the hedgehog is represented by the ‘hedgehog’ in the figure. Results show that the 28 mitochondrial proteins are well classified according to their biological orders. The proteins are classified into two main groups, i.e., the group of primates and the group of other mammals.

For the primates, the proteins are classified according to their families and subfamilies. The Ponginae subfamily (Sumatran orangutan and Bornean orangutan, *c* = 0.82) and the Homininae subfamily (Pygmy chimpanzee and Chimpanzee, *c* = 0.84) are grouped together with Gibbon (Hylobatidae family) to form a larger group. The Hominoidea family (Gorilla and Human, *c* = 1) is first clustered with the Hedgehog (Eulipotyphla order) and separated from all the other mammals but eventually joins the whole network when the threshold significantly decreases.

For non-primate mammals, the proteins are classified into different biological orders (Carnivora, Perissodactyla, Artiodactyla, Marsupialia, and Rodentia). The proteins of the Carnivora order are classified into the Phocidae family (Gray seal and Harbor seal, *c* = 0.9), Canidae family (Dog and Wolf, *c* = 0.9), Ursidae family (Brown bear, Polar bear, and Black bear, *c* = 0.89), and Felidae family (Cat and Tiger, *c* = 0.88), in which the first three families (Phocidae, Canidar, Ursidae) are closely related. The proteins of Perissodactyla are classified into the Equidae family (Horse and Donkey, c = 0.9) and the Rhinocerotidae family (Rhinoceros). The Carnivora, Perissodactyla and Artiodactyla (Cow, Platypus) form the core of the non-primate group, where the remaining (non-primate) species (Marsupialia: Opossum and Wallaroo; Cetacea: Fin whale and Blue whale; Rodentia: Mouse and Rat) are clustered around it.

To compare with machine learning methods, PseAAC features [33] are extracted from the sequences and libSVM [15] is applied to classify the proteins. In libSVM analysis, the 28 mammal species are classified into different biological orders. Gorilla, Human, and Bornean orangutan, which belong to the Primate order, are mis-classified to other mammal orders by the libSVM. Black bear, which is in Carnivora order, is mis-classified into Cetacea order. Rhinoceros, a species in Perissodactyla order, Platypus and Cow, species in Artiodactyla order, Mouse, belonging to Rodentia order, are all mis-classified. The remaining species are correctly classified. The results of libSVM clearly figure out whether a species belongs to an order or not, although mis-classifications may occur. The mammal orders identified by libSVM are partly consistent with our classification results, but libSVM doesn’t able to explain the relationships between the different biological orders.

### Mitochondrial proteins of 35 mammal species

The second example is a set of 35 NADH dehydrogenase encoded by the mitochondrial genes from 35 different mammal species [13]. The data were originally used in [13] for genome classification. The GenBank accession numbers of the 35 mammal genes are given as follows [13]: Human (V00662), Pygmy chimpanzee (D38116), Common chimpanzee (D38113), Gorilla (D38114), Gibbon (X99256), Baboon (Y18001), Vervet monkey (AY863426), Ape (NC 002764), Bornean orangutan (D38115), Sumatran orangutan (NC 002083), Cat (U20753), Dog (U96639), Pig (AJ002189), Sheep (AF010406), Goat (AF533441), Cow (V00654), Buffalo (AY488491), Wolf (EU442884), Tiger (EF551003), Leopard (EF551002), Indian rhinoceros (X97336), White rhinoceros (Y07726), Harbor seal (X63726), Gray seal (X72004), African elephant (AJ224821), Asiatic elephant (DQ316068), Black bear (DQ402478), Brown bear (AF303110), Polar bear (AF303111), Giant panda (EF212882), Rabbit (AJ001588), Hedgehog (X88898), Norway rat (X14848), Vole (AF348082), and Squirrel (AJ238588).

**Fig 4:**
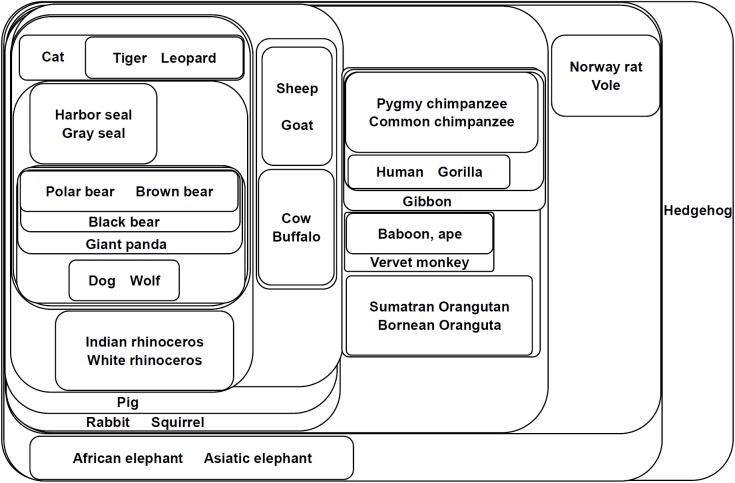
Component graph of mitochondrial proteins of 35 mammal species. This figure shows the connected components of 35 mitochondrial proteins of different mammal species. Each set represents a connected component obtained from the adjacency matrix filtered by a certain threshold (*T*_*c*_ = *c*•*A*_*max*,_ *c*∈[0,1]). Components with a higher threshold are included with the components with a lower threshold. The proteins are represented by their mammal species.

The classification results of the 35 NADH dehydrogenase are shown in Fig 4, where the proteins are represented by their mammal species. In this figure, the NADH dehydrogenase are classified according to their orders (Carnivora, Artiodactyla, Perissodactyla, Lagomorpha, Rodentia, Proboscidea, Primate, and Eulipotyphla). Among these orders, there are four main clusters: Primate, Rodentia (Norway rat, Vole), Eulipotyphla (Hedgehog), and other non-primate orders.

For the primates, the NADH dehydrogenase are classified into three small groups. The first group is the Homininae family (Pygmy chimpanzee, Common chimpanzee, Gorilla and Human, *c* = 0.85); the second group is the Cercopithecidae (baboon, Vervet monkey) and Hominidae (ape) families; and the third group is the Hominidae family (Ponginae: Sumatran orangutan and Bornean orangutan, *c* = 0.8).

The non-primate orders are also well classified. The Carnivora order is classified into Phocidae (Gray seal and Harbor seal, *c* = 0.98), Ursidae (Brown bear, Polar bear, Black bear and Giant panda *c* = 0.94), Canidae (Dog and Wolf, *c* = 1), and Felidae (Cat and Tiger, Leopard, *c* = 0.94). Proteins of the Perissodactyla order (Indian rhinoceros and White rhinoceros, *c* = 0.93) are also well grouped. Moreover, proteins of the Artiodactyla are classified into the Bovinae subfamily (Cow and Buffalo, *c* = 0.97) and the Caprinae subfamily (Sheep and Goat, *c* = 0.96), as well as the Suidae family (Pig). The rabbit, a separate species, belongs to the Lagomorpha order.

Among the many non-primate orders, the Carnivora order is the core of other species, which are enclosed by the Perissodactyla order and the Artiodactyla order.

The libSVM also classifies the 35 mammal species into different biological orders. Most of the species are correctly classified, with a few exceptions. Sumatran orangutan and Bornean oranguta, which are primates, are both mis-classified into the Artiodactyla order. All the three species (Norway rate, Vole, Squirrel) in the Rodentia order are wrongly classified. Also, Sheep and Pig, belonging to the Artiodactyla order, Dog and Cat, in the Carnivora order, are all improperly classified. The mammal classes that are correctly classified by libSVM are consistent to the clusters found by our method.

### Beta-globin of 50 animal species

The third example is a set of beta-globins of 50 different animal species. The data were originally used in [8, 11] for protein classification analysis; the accession numbers of these beta-globins are as follows: Human (AAA16334.1), Pigeon (P11342.1), Goshawk (P08851.1), Black bear (P68012.1)), Lesser panda (P18982.1), Asiatic elephant (P02084.1), Giant panda (P18983.2), African elephant (P02085.1), Sheep (P02075.2), Tortoise (P83123.3), Duck (P02114.2), Grivet (P02028.1), Mallard (P02115.1), Gorilla (P02024.2), Goose (P02117.1), Shark (P02143.1), Rat (CAA33114.1), Hippopotamus (P19016.1), Penguin (P80216.1), Horse (P02062.1), Swift (P15165.1), Gibbon (P02025.1), Coyote (P60525.1), Whale (P18984.1), Catfish (O13163.2), Bat (P24660.1), Bison (P09422.1), Red fox (P21201.1), Swan (P68945.1), Marmot (P08853.1), Buffalo (P67820.1), Salmon (Q91473.3), Dog (P60524.1), Sparrow (P07406.1), Chimpanzee (P68873.2), Pheasant (P02113.1), Dolphin (P18990.1), Flamingo (P02121.1), Goldfish (P02140.1), Pig (P02067.3), Polar bear (P68011.1), Dragonfish (ADD73488.1), Rhinoceros (P09907.1), Parakeet (P21668.1), Chicken (P02112.2), Zebra (P67824.1), Wolf (P60526.1), Cod (O13077.2), Turtle (P13274.1), and Langur (P02032.1).

**Fig 5:**
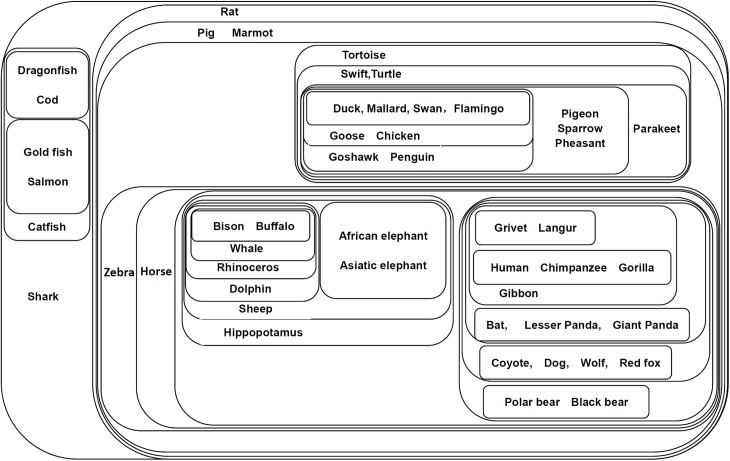
Component graph of 50 beta-globins of different animal species. This figure shows the connected components of 50 beta-globins of different animal species. Each set represents a connected component obtained from the adjacency matrix filtered by a certain threshold (*T*_*c*_ = *c*•*A*_*max*,_ *c*∈[0,1]). Components with higher thresholds are included with the components with lower thresholds. The proteins are represented by their animal species.

The classification results are shown in Fig 5. In this figure, the fish (Actinopterygii), avian, mammal and reptile species are well classified. These classification results are similar to those found by the protein map [8] and K-string dictionary method [11], with a few exceptions in the sub-classes.

In the large cluster of mammals, the beta-globins are divided into two groups, i.e., the group of Primate and Carnivora, and the group of Artiodactyla, Cetacea, Perissodactyla, and Proboscidea. In the group of Primates, the proteins are classified into different primate families: the Cercopithecidae family (Grivet, Langur), Hominidae family (Human, Chimpanzee, Gorrila), and Hylobatidae family (Gibbon). These Primate families are closely related to the Carnivora families: the Ursidae family (Lesser panda, Giant panda) and Canidae family (Coyote, Dog, Wolf, and Red fox). These mammal species form the main core of the protein network.

The beta-globins of avian species are also clustered. For all avian families presented, the Anatidae family (Duck, Mallard, Swan, *c* = 1) in biological order of Anseriformes is the core, which is enclosed by all other avian species.

The beta-globins of fishes are classified into two main groups according to their biological orders. The first group is the Actinopterygii class with the species Dragonfish, Cod, Goldfish, Salmon, and Catfish. Shark (Chondrichthyes class), which is separated from other species and stands alone as a second group.

From this analysis, we can see that mammals are the core of the network. Aves is the next class that encloses the mammals, followed by reptiles and, finally, fishes. Inside the group of mammals, the primates are the center for one of the mammal subgroups. Carnivora is the closest mammal order next to the primates. The other mammal orders, Artiodactyla, Cetacea, Perissodactyla, and Proboscidea, form another sub-group of the mammals.

The libSVM presents good classifications to the 50 beta-globins of animal species. The 4 big classes, namely, mammal, avian, fish, reptile, are all well-classified. Among the 50 beta-globins, only the beta-globins of Dragonfish, catfish, and turtle are wrongly classified, all the other 47 beta-globins are correctly identified. The 4 big classes identified by libSVM agree with the 4 main clusters identified by our method.

### HIV proteins

In the fourth example, we analyze the proteins of HIV. HIV (Human immunodeficiency virus) is a lenti virus that can lead to acquired immune deficiency syndrome (AIDS) [5, 13,34]. To develop anti-HIV drugs and vaccines, research into the origins and evolution of this virus is necessary. Rambaut et al. [5] used the maximum likelihood method to reconstruct a phylogenetic-tree for the primate lenti viruses, including HIV-1, HIV-2, and the simian immunodeficiency viruses (SIVs). It is discovered that the two HIV viruses have evolutionary origins in different SIVs. Here, we reuse their data to test on our new classification method. The data consisted of 33 proteins encoded from 33 different HIV and SIV genes. The RNA genomes are transformed into DNA sequences (change U to T) before being downloaded from the GenBank [5]. The sub-types of HIV-1, HIV-2 and SIV [5, 13], their primate hosts and the GenBank accession numbers are given as follows: HIV-1, group M: A (AF004885), B (A04321), C (AF443079), D (K03454), F (AY173957), G (AY772535), H (AF190127); group N (DQ017382); group O: A (AY169802), B (AY169803); HIV-2: A1 (AF082339), A2 (M30502), B1 (L07625), B2 (X61240); SIV chimpanzee (Pan troglodytes troglodytes): SIVcpz1 (AY169968), SIVcpz2 (AJ271369), SIVcpz3 (DQ373063); SIV chimpanzee (Pan troglodytes schweinfurthii): SIVcpz4 (DQ374657), SIVcpz5 (DQ374658); SIVdrl, drill (AY159321); SIVgsn, greater spot-nosed monkey (AF468659); SIVlhoest, L’ Hoest monkey (AF188114); SIVmac, macaque (D01065); SIVmnd1, mandrill (M27470), SIVmnd2, mandrill (AY159322); SIVmon, Campbells mona monkey (AY340701); SIVrcm, red-capped monkey (AF382829); SIVsab, Sabaeus monkey (U04005); SIVsm, sooty mangabey monkey (U72748); SIVsun, sun-tailed monkey (AF131870); SIVsyk, Sykes’ monkey (L06042); SIVtan, tantalus monkey (U58991); SIVver, vervet monkey (M29975).

**Fig 6:**
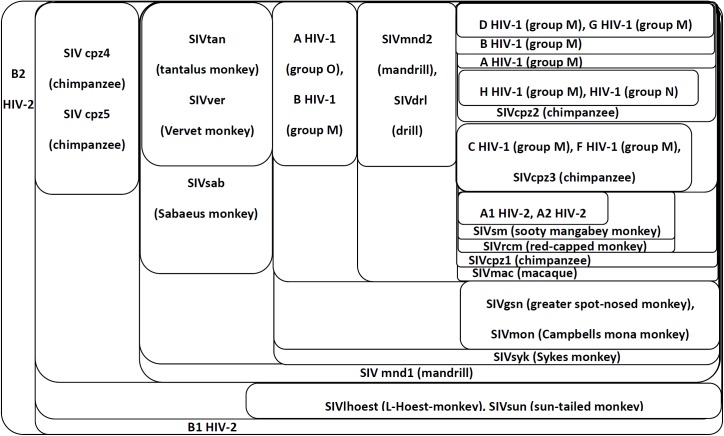
Component graph of HIV proteins. This figure shows the connected components of the HIV proteins. Each set represents a connected component obtained from the adjacency matrix filtered by a certain threshold (*T*_*c*_ = *c*•*A*_*max*,_ *c*∈[0,1]). Components with higher thresholds are included with components with lower thresholds. The proteins are labeled by their virus types (HIV-1,HIV-2, or SIV) and the hosts.

The classification results of these HIV proteins are shown in Fig 6. This figure shows that the HIV-1 and HIV-2 proteins have different lineages to the SIV proteins. The proteins of HIV-1 group M are closest to the proteins of SIV, chimpanzee (Pan troglodytes troglodytes). The proteins of HIV-2 A2 and A1 are closely related to the proteins of SIVsm (sooty mangabey monkey), SIVrcm (red-capped monkey), SIV chimpanzee (Pan troglodytes troglodytes) and the SIVmac (macaque). The proteins of HIV-1 and HIV-2 A are closely related and have close relations with some common SIV proteins. However, the proteins of HIV-2 B are far away from the proteins of HIV-1 and HIV-2, which have different lineages to SIV proteins. In this analysis, the proteins of HIV B1 and HIV B2 are closely related to SIV chimpanzee (Pan troglodytes schweinfurthii) and SIVlhoest (L’ Hoest monkey) and SIVsun (sun-tailed monkey). These classification results are similar to the results of the maximum likelihood method [5] but are slightly different from the results of the moment vectors [13].

The libSVM presents many mis-classifications for the HIV-1, HIV-2, and SIV proteins. There are 4 out of 10 HIV-1 proteins are wrongly classified to the class of SIV. All 4 proteins in the HIV-2 class are mis-classified to the class of SIV. Also, in the class of SIV, 5 out of 19 proteins are wrongly classified to the class of HIV-1. Even though, the HIV-2 proteins are mis-classified to the SIV class, but libSVM doesn’t tell which SIV proteins have close lineages to these HIV-2 proteins. Also, the mis-classification of some SIV proteins into the class of HIV-1 proteins, may imply that these SIV proteins are closely related to the HIV-1 proteins, but the results are quite different from those close lineages found by our method. The libSVM merely does yes/no classifications which doesn’t pinpoint the relations between classes. The libSVM doesn’t work well for this example.

### Influenza A virus

The Influenza A virus is a kind of negative-sense, single-stranded, segmented RNA virus [6]. Here, we analyze the set of 52 proteins encoded by the genes of influenza A virus [6]. These influenza viruses are specified by three characters: the virus sub-type, the geographical location of the case and the host of the influenza A virus. The influenza A virus has many different sub-types; an H number indicates the type of hemagglutinin, and an N number indicates the type of neuraminidase. The dataset consists of six virus sub-types: H7N3, H11N9, H1N1, H7N9, H3N2, H5N1 [6].

**Fig 7:**
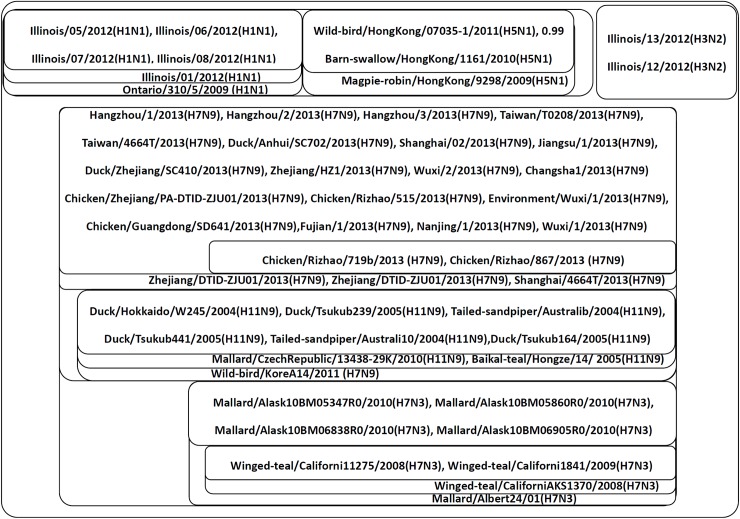
Component graph of the Influenza A virus proteins. This figure shows the connected components of the Influenza A virus proteins. Each set is a connected component obtained from the adjacency matrix filtered by a certain threshold (*T*_*c*_ = *c*•*A*_*max*,_ *c*∈[0,1]). Components with higher thresholds are included with components with lower thresholds. The proteins are labeled by their host, geographical location, year of the case and influenza A virus subtype.

The classification results are shown in Fig 7. In this figure, the proteins are clearly classified according to influenza A virus sub-type. Within each sub-type, the proteins are classified in terms of their host and geographical location. For instance, the proteins of H7N3 are mainly classified into two groups and one separate protein, namely, the group of Alaska with Mallard as the host, the group of California with Winged-teal as the host, and an individual protein of Alberta with Mallard as the host.

Among the different virus sub-types, proteins are first classified by their neuraminidase types, i.e., the N number. In this example, the proteins are first classified into three main groups: the group of H1N1 and H5N1, the group of H3N2, and the group of H7N3, H7N9 and H5N9. The group of HIN1 and H5N1 are classified into two subgroups, namely, the subgroup of HIN1 and the subgroup of H5N1. The group of H7N3, H7N9 and H11N9 is first classified into a subgroup of H7N9 and H11N9 and the subgroup of H7N3. Then, the subgroup of H7N9 and H11N9 is further classified into subgroup H7N9 and subgroup H11N9.

These results imply that the influenza A viruses are first classified by their neuraminidase type (N number) and then by their hemagglutinin type (H number). In other words, proteins of the same neuraminidase type form a general class, which is further divided into sub-classes of different hemagglutinin types. Moreover, within each virus subtype, the proteins are further classified by their geographical location and host.

The libSVM also correctly identified the six influenza A virus subtypes. All H7N3, H1N1, H3N2 and H5N1 proteins are correctly classified, and majority of the H11N9 and H7N9 proteins are properly identified, except for 1 protein of H11N9 is mis-classified to the class of H7N9, and 1 protein of H7N9 is mis-classified to the class of H11N9. However, libSVM doesn’t have the abilitly to identify the relationships among the six virus subtypes, and hence cannot reveal the relationships of the neuraminidase types and hemagglutinin types.

### Protein kinase C

In the sixth example, we analyzed the protein kinase C families. Protein kinase C (PKC) is a family of enzymes involved in controlling the function of other proteins through the phosphorylation of hydroxyl groups of serine and threonine amino acid residues on these proteins [10]. The entire PKC family can be divided into six subfamilies: cPKC, nPKC, aPKC, PKC*μ*, PKC1 and PRK. There are 124 proteins in the set; descriptions and GenBank accession numbers are fully listed in the appendix of [10].

**Fig 8:**
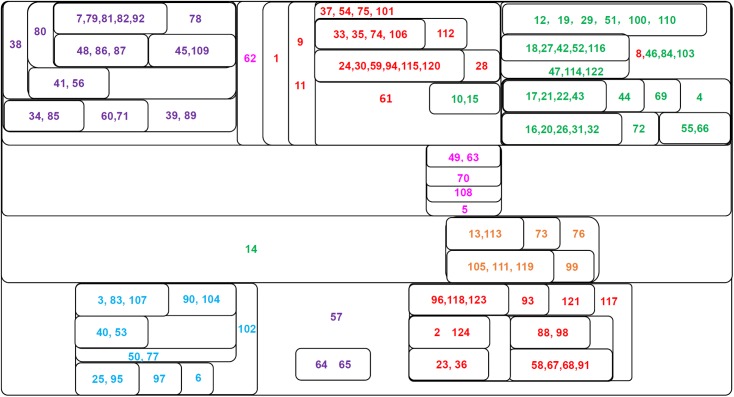
Component graph of the PKC families. This figure shows the connected components of the PKC (protein kinase C) at different thresholds. Each set stands for a connected component obtained by the adjacency matrix filtered by a certain threshold (*T*_*c*_ = *c*•*A*_*max*,_ *c*∈[0,1]). Components with higher thresholds are included with components with lower thresholds. Each PKC is labeled by a unique integer between 1 and 124, corresponding to the protein index as referenced in [10]. Different PKC families are highlighted by different colors: aPKC (blue), cPKC (green), nPKC (red), PKC1 (purple), PRK (pink), PKCμ (orange). A detailed description of the PKCs can be found in the supplementary materials of [10].

The classification results of these PKC families are shown in Fig 8. In this figure, each protein is labeled by a unique integer from 1 to 124 corresponding to the index of the protein given in the table of [10]. From this figure, we can see that the six PKC subfamilies are clearly classified: PKC1 (upper left block, purple), nPKC (upper middle and bottom right blocks, red), cPKC (upper right block, green), PRK (center block, pink), PKC*μ* (below center block, orange), and aPKC (bottom left block, blue). Proteins from the same PKC subfamilies are highlighted with the same colors.

Proteins of the same PKC subfamilies are gathered in the same blocks. Within each block of the same PKC subfamily, the proteins are further classified in terms of their NCBI descriptions. The nPKCs are divided into different sub-groups: *η* (33, 35, 74, 106, 112), (24, 30, 59, 94, 115, 120, 28), *δ* (1, 2, 124, 23, 36, 88, 98, 58, 67, 68, 91), and Serine\Threonine (9, 11), *θ* (96, 118, 123, 93, 121). The cPKCs are also divided into subgroups of *γ* (18, 27, 42, 52, 116, 47, 114, 122), *α* (16, 20, 26, 31, 32, 72), and *β* (17, 21, 22, 43, 44, 69). The aPKCs are classified into *ι* (3, 83, 107, 90, 104, 40, 53) and *ζ* (25, 95, 97, 6). The PKCμ proteins are divided into subgroups of ν (13, 113, 73) and μ (105, 111, 119, 99). The classification results (i.e., the groups) are similar to those branches found by the natural vector method [11] and the classes identified by libSVM. In the libSVM analysis, the six PKC subtypes are correctly identified, except for 6 proteins that are mis-classified.

The difference between our results and the natural vector results is that our results are based on the multivariate connection rather than bivariate classification of proteins. In our analysis, proteins in the same connected component are assumed to be connected. Among all PKCs, the three subfamilies, PKC1 (purple), cPKC (green) and the η, ε and the Serine\Threonine sub-classes of the nPKC (red) are the most closely related than the other subfamilies. PRK (pink) is the next subfamily enclosed around them (with weaker connections), followed by the PKCμ (orange) and aPKC (blue) subfamilies. The *δ* and *θ* sub-classes of nPKC (red) are the farthest groups and have the weakest connections to the rest.

### Protein structures of 3 CATH groups

In the seventh example, we do the structural classification of 3 CATH groups. CATH is a protein structural database, where proteins are classified by their Classes (C), Architecture (A), Topology (T) and Homologous superfamily (H). In this example, we use three CATH groups to demonstrate the application of our method in 3D structures.

The 3 CATH groups are from 3 different classes, Class 1: group 1.10.220 (mainly alpha structures, C = 1, A = 10, T = 220), contains 14 proteins in total; Class 2: group 2.60.20 (mainly beta structures, C = 2, A = 60, T = 20), contains 12 proteins in total; Class 3: group 3.10.310 (mixed alpha and beta structures, C = 3, A = 10, T = 310), contains 10 proteins in total. The PDB ID of these structures can be found in official website of CATH.

To classify the structures, protein 3D coordinates are converted to (φ,ψ) torsion angles by using ‘ramachandran’ function in matlab. Each protein structure is converted to a list of (φ,ψ) torsion angles. The mutual information rates are computed on the real valued torsion angles.

**Fig 9:**
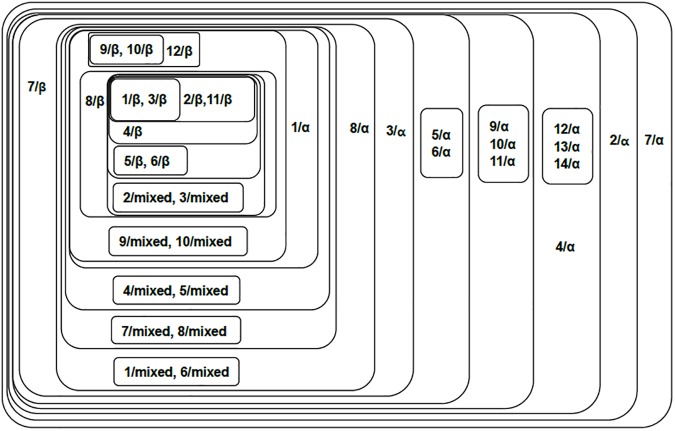
Component graph of 3 CATH groups. This figure shows the connected components of 3 CATH groups at different thresholds. Each set stands for a connected component obtained by the adjacency matrix filtered by a certain threshold (*T*_*c*_ = *c*•*A*_*max*,_ *c*∈[0,1]). Components with higher thresholds are included with components with lower thresholds. The proteins are labeled by their index and classes (α represents group 1.10.220, mainly alpha helix, Class 1; β represents group 2.60.20, mainly beta sheets, Class 2; ‘mixed’ represents group 3.10.310, mixed alpha and beta).

The classification results of the 3 CATH groups are shown in Fig 9. In this figure, we can see that the three structural classes (alpha, beta, and mixed) are well-classified. The mainly beta structures form the core of the network, with mixed alpha and beta structures enclose around, mainly alpha structures locate at the peripheral of the network. There are also a few exceptions with the alpha/beta classification. For instance, the 2nd and 3rd proteins of the mixed structures are shown to be closely related to some beta structures, this may be because the mixed structures are the mixtures of the alpha and beta structures, and the beta parts of these mixed structures are closely related to the mainly beta structures.

The libSVM does not perform well in this example. In the class of mainly alpha structures, 9 out of 14 proteins are mis-classified as mainly beta structures, and 2 out of 14 proteins are wrongly classified as mixed alpha and beta structures. In the class of mainly beta structures, 3 out of 12 proteins are mis-classified as mainly alpha structures, another 3 out of 12 proteins are mis-classified as mixed alpha and beta structures. Moreover, in the class of mixed alpha and beta structures, 3 out of 10 proteins are mis-classified as alpha structures, while 6 out of 10 proteins are mis-classified as beta structures. Among all 36 proteins, only 10 proteins are correctly classified.

### Protein sequences from Rhodopsin Like/Peptide family

The eighth example comprises two sub-families (Chemokine and Melanocortin) of proteins belonging to the Rhodopsin Like/Peptide family [35]. The dataset contains 510 protein sequences in total, 255 proteins from each sub-family. To compare with machine learning methods, the whole dataset is split into training data (480 proteins, 240 proteins from each sub-family) and test data (30 proteins, 15 proteins from each sub-family). The protein data can be found in the supplementary material of [35].

**Fig 10:**
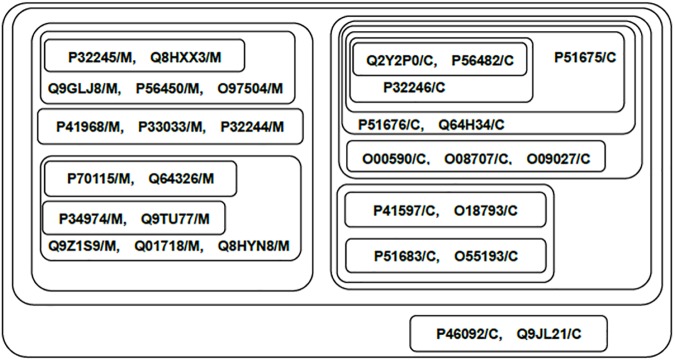
Component graph for proteins of two Rhodopsin Like/Peptide sub-families. This figure shows the connected components of two Rhodopsin Like/Peptide sub-families at different thresholds. Each set stands for a connected component obtained by the adjacency matrix filtered by a certain threshold (*T*_*c*_ = *c*•*A*_*max*,_ *c*∈[0,1]). Components with higher thresholds are included with components with lower thresholds. The proteins are represented by their NCBI accession number and subfamilies (C: Chemokine, M: Melanocortin).

The classification results are shown in Fig 10. We can see that the Chemokine and Melanocortin subfamilies are clearly classified. The proteins of the same subfamilies are closely related, while proteins of different subfamilies are separated. LibSVM [15] also correctly identifies the two subfamilies.

### Protein sequences from 4 structural classes

The ninth example consists of 277 proteins from 4 structural classes in SCOP [35,36]. The class 1 (all-α domains) contains 70 proteins, the class 2 (all-β domains) contains 61 proteins, the class 3 (α/β domains, α and β proteins: Mainly parallel beta sheets, beta-alpha-beta units) contains 81 proteins, the class 4 (α+β domains, α and β proteins: Mainly antiparallel beta sheets, segregated alpha and beta regions) contains 65 proteins. To compare with machine learning methods, 9/10 of the whole data is used as training data, and the remaining are used as test data. The test data comprises of 27 proteins, 7 from the all-α class, 6 from the all-β class, 8 from the α/β class, and 6 from the α+β class. The sequence file of these proteins can be found in the supplementary material of [35].

**Fig 11:**
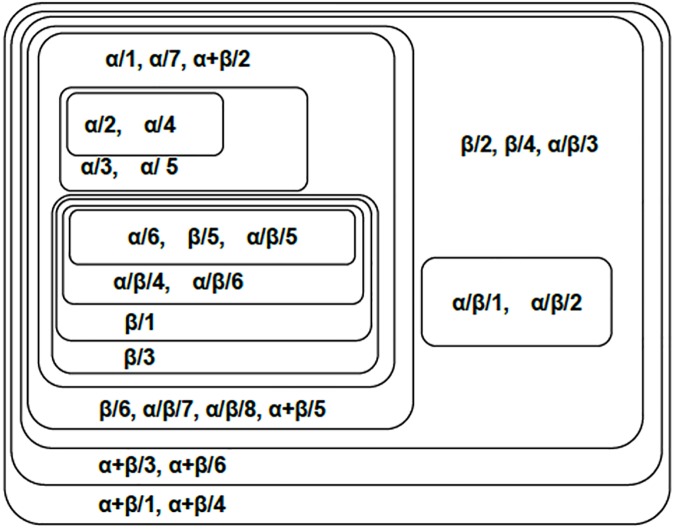
Component graph of the 4 SCOP classes. This figure shows the connected components of 4 SCOP classes at different thresholds. Each set stands for a connected component obtained by the adjacency matrix filtered by a certain threshold (*T*_*c*_ = *c*•*A*_*max*,_ *c*∈[0,1]). Components with higher thresholds are included with components with lower thresholds. The proteins are labeled by their SCOP classes and index of the proteins in their classes.

The classification results are shown in Fig 11. In this figure, proteins of the same classes are closely clustered. The all-α class presents as the core of the network, i.e. mutually connected with the strongest relations. The all-β class is next to the core, which is enclosed by the α/β and the α+β classes. Note in this figure that the all-β class is closely related to the α/β and α+β classes. This makes senses, because both α/β and α+β classes are the mix of alpha and beta proteins, where α/β is for mainly parallel beta sheets (beta-alpha-beta units), while α+β is for mainly antiparallel beta sheets (segregated alpha and beta regions).

In the libSVM analysis, majority of the proteins are correctly classified. In the all-α class, all 7 proteins are correctly identified. Also, 3 out of 6 proteins in the all-β class, 7 out of 8 proteins in the α/β class, and 5 out of 6 proteins in the α+β class are properly classified. However, there is a main difference between the machine learning methods e.g. libSVM and our method that, machine learning methods do yes/no classifications that divide the solution space into disjoint classes, but our method reveals the mutual relations between proteins and identifies the clusters according to their connections.

## Discussion

In this paper, we use the theory of information and networks to develop a new approach to protein evolutionary classification. We have used nine examples to verify our method. Results show that the new method is not only efficient but also interprets the protein relationships from a new perspective.

We model the protein universe as an undirected network and classify proteins according to their strength of connections. In network theory, the connected components represent a gathering of nodes where all members are mutually connected. To take advantage of this property, we classify the proteins into different components. Each component represents a class of proteins whose members are strongly connected to each other. In the same connected component, the mutual relations are stronger for proteins within the same sub-component than proteins from a different sub-component. Typically, the core component in the center of a network has the strongest mutual relations among its proteins.

The performance of protein classifiers is usually influenced by sequence similarity [16]. Our method, that is based on mutual information rates, is also influenced by sequence similarity. It is believed that protein sequence is related to its structure and function. In consequence, protein sequence similarity is also a key factor to identify the similarity of structures and functions. Many unsupervised classifiers particularly take advantages of this fact to classify proteins [2–4,6–14]. For our method, the protein data are selected under the same conditions such that each protein is a unique representative of an animal species or a virus subtype in the dataset, the homology of protein sequences reflects only the evolutionary similarity of different animal species or virus subtypes. If the sequence identity is below a threshold of e.g. 30%, our method should still work. Because, as defined for the connected components, the filtering threshold is adjustable to the overall magnitudes of the adjacency matrix and is independent to specific similarities. Our method do care about the relations among all proteins rather than specific pair. The similarity of individual proteins doesn’t have determinant impact on our results, because the similarity between each pair of proteins is compared to all pairs, and the connected components are obtained by adjusting to situation of the whole network.

The main classification groups found by our method are similar to those tree-branches identified by phylogenetic-trees. With respect to the animal species, the proteins are classified into groups that correspond to different biological orders. Within each group, the proteins are further classified into subgroups consistent with their biological subfamilies. The classification results of the 35 mammal species proteins are in conformance with the classification results of the moment vectors [8] and libSVM, although the sub-groups that should be classified pair by pair using polygenetic-trees are multi-classified using our method.

For mammal species, Carnivora (biological order) is found to be the core of the non-primate species, and Perissodactyla and Artiodactyla are the closest biological order next to Carnivora. In the beta-globin analysis, the primates present as one of the centers of mammals, where the other center is formed by Perissodactyla and Artiodactyla. The clusters identified by our method are consistent to the branches presented by polygenetic-trees [8, 11] and the classes identified by libSVM. Here, Carnivora is the closest biological order next to the primates.

For the HIV proteins, the proteins of HIV-1 are close to the proteins of HIV-2 A, whereas the proteins of HIV-2 B are far away from the proteins of HIV-1 and HIV-2 A. This implies that the HIV-1 and HIV-2 A proteins share common lineages with SIV proteins. This outcome consists of the classification results found by the maximum likelihood method [5] but is slightly different from the results of the moment vectors [13]. LibSVM doesn’t identify any sensible relation between HIV-1, HIV-2 and SIV proteins.

For the classification of the influenza A virus, our method outperforms the Yau-Hausdorff distance and libSVM. In our analysis, the classifications are clear and reasonable; the proteins are classified first by their virus subtypes and then by their geological locations and virus hosts. The Yau-Hausdorff distance [6] does not present sensible classification results in this case. In addition, based on our analysis, we found that the neuraminidase type, i.e., N number, is the first index to classify the proteins; it decides the general groups of the proteins, after which the proteins are further classified by their hemagglutinin type, i.e., the H number. LibSVM cannot figure out this point. The results strongly demonstrate the efficiency of our new method.

In PKC analysis, the proteins are clearly classified into different PKC subfamilies. Within each PKC subfamily, the proteins are further classified into groups that correspond to their NCBI descriptions.

For the 3 CATH groups, the three structural classes are well-classified by our method, whose clusters are similar to the classes found by the libSVM. This analysis shows the practicality of our method in 3D structural classification. The good performances of our method in the Rhodopsin Like/Peptide family and SCOP data analysis additionally verify the usefulness of our method.

Our method has many good properties. It is unsupervised, alignment free, and can be applied to both sequences and 3D structures.

Machine learning methods such as libSVM [15] are supervised classification methods that depend on pre-labeled training data, and web servers e.g. PseAAC [33] and Pse-in-One [37] are required to extract protein features before analysis. Our method is unsupervised, which does not require the usage of training data and feature extraction process. Therefore, our method is particularly useful in protein evolutionary classifications, where the classes of proteins are usually unknown.

Machine learning classifiers usually do yes/no classifications that split the solution space into disjoint classes. Unlike these methods, our method analyzes the relationship of proteins and classify proteins according to their relations. For instance, in the HIV analysis, the HIV-1 and HIV-2 proteins are found to have different lineages to the SIV proteins by using our method, but libSVM sharply divides the proteins into three classes (HIV-1, HIV-2, and SIV) without tagging their lineages.

Our method is independent of the alignment of sequences. This is because, the maximum mutual information rate is an information quantity, which relies on only the probability distribution of amino acids and does not require the alignment of protein sequences.

Our method can be applied to both sequences and structures. Protein 3D structures can be converted to torsion angles and applied with our method by computing the mutual information rates on torsion angles. The applicability of our method on both protein sequences and 3D structures is a key advantages over other methods, because early methods such as K-string dictionary [11], protein map [8], natural vectors [10, 14], and Yau-Hausdorff distance [6], can be applied on either but not both.

Another key feature of our new method is that it models the protein universe as a network and solves the classification problem by using connected components. Our method takes the most of information and network tools to identify the multivariate relationships of proteins. By virtue of connected components, all members of the same component are mutually connected. Protein classification can be represented by the inclusion and exclusion of these connected components. This is also a main difference between our method and other existing methods.

## Conclusion

In this paper, we have introduced a novel approach on protein classification. The new method is based on the theory of information and networks; it models the protein universe as undirected networks and analyzes the multivariate relationships of proteins. Our method is an efficient and alignment-free method in the classification of proteins and may have wide applications to both sequences and structures.
